# Intraoperative MRI-derived volumetric ablation margins and initial correlation with local outcome after MRI-guided cryoablation of renal tumors

**DOI:** 10.1186/s40644-023-00546-x

**Published:** 2023-03-30

**Authors:** Nienke S. de Jager, Tim J. van Oostenbrugge, Torben Pätz, Sjoerd F. M. Jenniskens, Jurgen J. Fütterer, Johan F. Langenhuijsen, Christiaan G. Overduin

**Affiliations:** 1 Department of Medical Imaging, Radboud University Medical Center, Geert Grooteplein Zuid 10, P.O. Box 9101, 6525 Nijmegen, GA Netherlands; 2 Department of Urology, Radboud University Medical Center, Geert Grooteplein Zuid 10, 6525 Nijmegen, GA Netherlands; 3 Fraunhofer Institute for Digital Medicine, Max-von-Laue-Str. 2, 28359 Bremen, Germany

**Keywords:** Renal tumor, Percutaneous cryoablation, MRI, Treatment margin, Local recurrence

## Abstract

**Purpose:**

To assess volumetric ablation margins derived from intraoperative pre- and post-ablation MRI after magnetic resonance imaging (MRI)-guided percutaneous cryoablation of renal tumors and explore its correlation with local treatment success.

**Methods:**

Retrospective analysis was performed on 30 patients (mean age 69y) who underwent percutaneous MRI-guided cryoablation between May 2014 and May 2020 for 32 renal tumors (size: 1.6–5.1 cm). Tumor and ice-ball volumes were segmented on intraprocedural pre- and post-ablation MR images using Software Assistant for Interventional Radiology (SAFIR) software. After MRI-MRI co-registration, the software automatically quantified the minimal treatment margin (MTM),defined as the smallest 3D distance between the tumor and ice-ball surface. Local tumor progression (LTP) after cryoablation was assessed on follow-up imaging.

**Results:**

Median follow-up was 16 months (range: 1–58). Local control after cryoablation was achieved in 26 cases (81%) while LTP occurred in 6 (19%). The intended MTM of ≥5 mm was achieved in 3/32 (9%) cases. Median MTM was significantly smaller for cases with (− 7 mm; IQR:-10 to − 5) vs. without LTP (3 mm; IQR:2 to 4) (*P* < .001). All cases of LTP had a negative MTM. All negative treatment margins occurred in tumors > 3 cm.

**Conclusions:**

Determination of volumetric ablation margins from intraoperative MRI was feasible and may be useful in predicting local outcome after MRI-guided renal cryoablation. In our preliminary data, an intraoperative MRI-derived minimal margin extending at least 1 mm beyond the MRI-visible tumor led to local control and this was more difficult to achieve in tumors > 3 cm. Ultimately, online margin analysis may be a valuable tool to intraoperatively assess therapy success, but larger prospective studies are needed to establish a reliable threshold for clinical use.

## Introduction

Kidney cancer represents 2–3% of all cancers, with renal cell carcinoma (RCC) accounting for 90% of all renal cancers [1]. During the last two decades, RCC incidence rates worldwide have been increasing with an annual trend of 2% [2]. This trend is due to the widespread use of abdominal imaging, leading to an increased detection of incidental findings [3]. Of all RCC diagnosis, 60–70% are these so-called incidentalomas which are often classified as small renal tumors [1].

The standard of care for early stage renal tumors (T1a-b) is a partial nephrectomy if technically feasible [2]. Alternative treatments can be considered based on patient preference or in several specific patient groups such as elderly, those with multiple morbidities, bilateral tumors, hereditary RCC syndromes and limited renal functional reserve due to renal insufficiency or a solitary kidney [4]. These alternative treatments include minimally invasive ablative therapies such as percutaneous cryoablation (PCA) [2].

PCA of renal tumors under magnetic resonance imaging (MRI)-guidance is safe and feasible [5, 6]. Potential advantages of MRI-guidance compared to standard computed tomography (CT)-guidance are the native high soft-tissue contrast, the capability of direct multiplanar imaging, the lack of ionizing radiation and the ability of near real-time monitoring of the full ice ball volume progression, allowing a volumetric assessment of tumor-ice coverage [6, 7]. Experimental studies have shown that temperatures of − 20 °C [8] and − 40 °C [9] are necessary to ensure complete cell death in healthy renal parenchyma and in tumor cells, respectively. Animal studies have shown that these temperatures can be achieved if the ice ball extends beyond the tumor border with at least 3 mm for − 20 degrees [10] and 5–6 mm for − 40 degrees [11]. Based on these studies, most interventionalists aim for a treatment margin of 5 mm to minimize the risk for local recurrent disease [10, 12–14]. However, recent studies have indicated that renal tumors may be treated successfully with smaller treatment margins of at least 1–1.5 mm [15, 16]. Nevertheless, studies investigating the optimal treatment margin are scarce and these are often limited to two-dimensional measurements or ablation zone delineation on post-interventional CT or MRI data and therefore do not allow an intraoperative assessment of the actual achieved treatment margin.

The objective of the present study was to assess feasibility of retrospectively deriving volumetric ablation margins from intraoperative pre- and post-ablation MR imaging after magnetic resonance imaging (MRI)-guided percutaneous cryoablation of renal tumors and explore its correlation with local treatment success.

## Materials and methods

Institutional Review Board approval for retrospective use of data was obtained for this single-center retrospective case study, and informed consent was waived. Data of several included patient data were previously reported in other studies [6, 17] that had different study objectives and were not focused on determining peri-ablational treatment margins.

### Patient selection

Between May 2014 and May 2020, a total of 37 patients with 39 small renal tumors who underwent MR-guided PCA at our institution were consecutively enrolled for this study. Exclusion criteria were absence of follow-up imaging at least 6 months after treatment, conclusive benign histopathology, technically failed ablation and patients refusing participation in scientific research. At our institution all renal cryoablation procedures are routinely performed under MRI guidance. Only patients contraindicated to undergo MR imaging (e.g. cardiac pacemaker, implant, or metal foreign object) or patients with a too large body circumference to enable percutaneous placement of the cryoprobes within the MRI bore are treated under computed tomography (CT) guidance, and were not included in this study. The decision to perform ablative therapy was made at the multidisciplinary tumor-board meeting. Ablative treatment was discussed for renal tumors < 4 cm in diameter suspicious for RCC based on diagnostic imaging, for which partial nephrectomy was not preferable due to comorbidities or by patient’s preference. Only if technically feasible, larger tumors (size > 4 cm) were also considered for treatment with ablative therapy. All patients in our institute selected for ablative therapy of a renal tumor are treated with cryoablation.

**Fig. 1:**
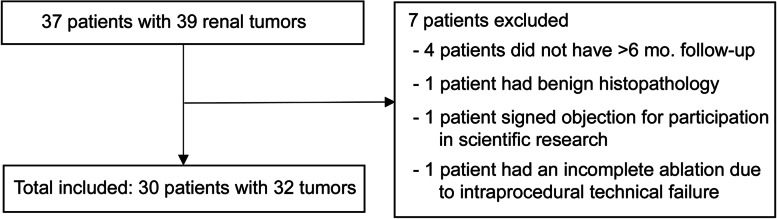
Schematic overview of patient selection

Patient selection is outlined in Fig. 1. The intraprocedural technical failure in one patient was due to an incidental malfunction of the cryoablation device. After exclusion, a total of 30 patients with 32 tumors were included in this study. Demographic characteristics are included in Table 1. One patient received retreatment of a local recurrence and one patient was treated in two separate sessions for bilateral tumors. Two patients had metastatic disease at the time of cryoablation. They both had a stable disease and did not receive systemic therapy at the time of the ablation, and therefore were not excluded in this study.

**Table 1:** Patient (*n* = 30) and tumor (*n* = 32) characteristics

	No (%), mean ± standard deviation (SD) or median (range)
Age (years)	69 (38–83)
Sex	
Male	20 (66.6)
Female	10 (33.4)
ASA classification	
II	17 (56.6)
III	13 (43.4)
Maximal tumor diameter (mm)	31.3 ± 9.4
Tumor side	
Left	16 (50.0)
Right	16 (50.0)
Tumor location	
Upper pole	7 (21.9)
Interpolar	11 (34.4)
Lower pole	14 (43.7)
Tumor site	
Anterior	13 (40.6)
Posterior	19 (59.4)
Histologic subtype	
Clear-cell RCC	14 (43.7)
Papillary RCC	3 (9.4)
Chromophobe RCC	2 (6.3)
Oncocytoma or mixed oncocytoma/chromophobe RCC	1 (3.1)
Inconclusive biopsy	7 (21.9)
No histopathology	5 (15.6)
Prior cryoablation	
First ablation	31 (96.9)
Re-ablation	1 (3.1)
RENAL score	
4–6 (low)	20 (62.5)
7–9 (moderate)	11 (34.4)
10–12 (high)	1 (3.1)

*ASA* American Society of Anesthesiologists

### Cryoablation procedures

All procedures were performed under general anesthesia by one urologist (J.L., 13 years of experience) and one of two interventional radiologists (J.F., S.J., with respectively 14 and 9 years of experience). At the start of the procedure, axial and coronal T2-weighted half fourier spin echo (HASTE; 1.2 × 1.2 mm in-plane resolution; 3.0 mm slice thickness) and axial T1-weighted stack-of-stars volume interpolated gradient echo (starVIBE; 1.0 × 1.0 mm in-plane resolution; 2.5 mm slice thickness) was performed as pre-treatment reference imaging. Subsequent ultrasound-guided core needle biopsy was performed in 26/32 procedures. BMI was not an exclusion criterion for ultrasound guided biopsy. Histological results from previous renal surgery were available in the other 6 patients. Multiple MR-compatible cryoprobes (IceSeed/IceRod, Galil Medical) were placed in the target lesion under ultrasound-guidance (Aplio i800, Canon Medical). The number and types of needles used were chosen at the physician’s discretion based on tumor size, location and manufacturer specifications. Probes were placed > 1 cm and < 2 cm from each other with the probe tips positioned at the most distal border of the tumour. Needle positions were verified using T2-weighted HASTE imaging and repositioned under MRI-guidance before the first freezing cycle if inadequate placement was suspected. All 32 procedures were performed in a wide-bore 3-T MR scanner, as previously described [17]. Cryoablation was performed using a double-cycle protocol of 10 min freezing, 2 min passive thawing and 1 min active thawing. Ice ball formation was monitored with continuous near real-time volumetric MR-imaging with either T1-weighted starVIBE or T2-weighted HASTE acquisitions. Ice ball coverage of the tumor was assessed by two dimensional side by side comparison during the procedure. We aimed for radiological complete coverage with an extension of 5 mm of the ice ball beyond the tumor border. In case of suspected incomplete coverage cryo-probe repositioning, placement of an extra probe or an additional third cycle was performed.

### Clinical outcome

All patients underwent routine follow-up imaging with contrast-enhanced MRI scans at 1, 3, 6, 12 and 18 months after cryoablation, followed by 1-year intervals. Follow-up scans were performed with contrast-enhanced CT scans in one patient due to an alternative follow-up schedule after a previous treatment with systematic treatment for metastasized RCC. Residual and recurrent disease were defined as nodular contrast enhancement in the ablation zone on the 1-month follow-up MRI-scan confirmed on subsequent follow-up imaging and on any follow-up imaging > 1 month, respectively. Local tumor progression (LTP) was defined as either residual or recurrent disease. Follow-up was recorded until December 2020 and ended 5 years after treatment, upon detection of LTP or upon death. The Clavien-Dindo Classification was used to record complications. Any grade ≥ 3 complication was considered a major complication. RENAL nephrometry score was applied and categorized as low (score 4–6), moderate (score 7–9) and high (score 10–12) [18]. Tumor location was classified as anterior when located for > 50% in the ventral half of the kidney or posterior when located for > 50% in the dorsal half of the kidney, based on pre-ablation axial imaging.

### Procedural imaging analysis

**Fig. 2:**
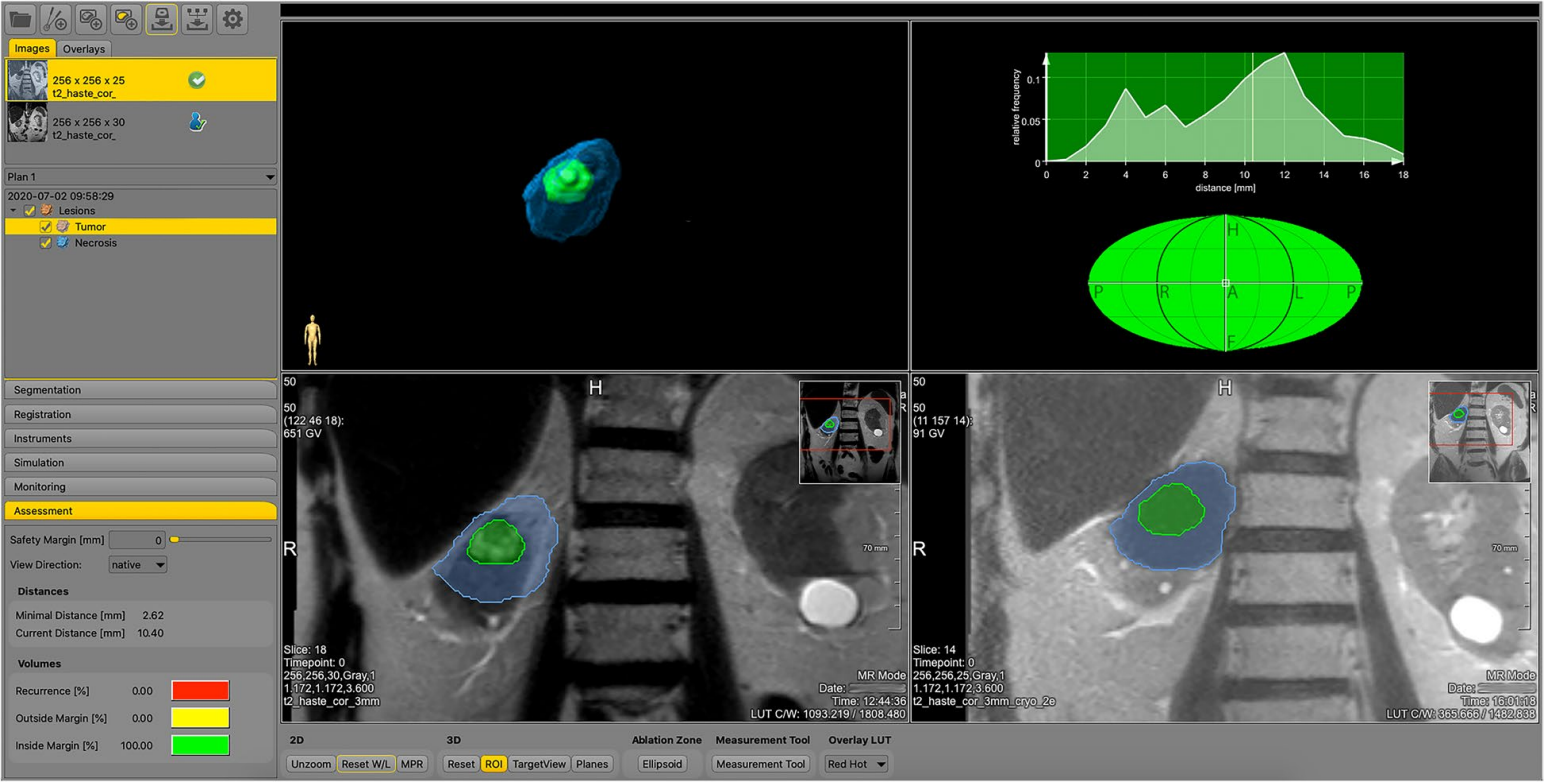
Overview of Software Assistant for Interventional Radiology (SAFIR) layout. Available image series and tools are shown in bars on the left. Image views from left to right, top to bottom: (1) 3D view of annotated ice ball (blue) and tumor (green) overlap, (2) tumor divided in octants along the left-right, cranio-caudal and anterior-posterior axes showing color-coded subareas of total tumor volume with complete coverage including user-defined treatment margin (green), complete coverage but insufficient margin (yellow) and incomplete coverage (red)., (3) pre-ablation and (4) post-ablation T2-haste coronal MRI-images showing the tumor (green) and co-registered ice ball (blue) volumes. Side bar on the left shows calculated 3D ablation margin metrics

A dedicated research software package (Software Assistant for Interventional Radiology (SAFIR) v1.3, Fraunhofer MEVIS, Germany) was used to retrospectively review intraprocedural MR-images (Fig. 2). Tumor and corresponding ice ball volumes were semi-automatically segmented on respectively pre-ablation and post-ablation intraprocedural T2-weighted HASTE or T1-weighted VIBE images. Semi-automatic segmentation consisted of an automatic interpolation focusing on the borders of the tumor or ice ball with manual correction if required. The pre-ablation and post-ablation images were initially registered automatically using a rigid image co-registration algorithm with manual adjustments to align the area of interest. Annotations and image co-registration accuracy were reviewed and if required corrected by an interventional radiologist (J.F.), who was blinded for the clinical outcomes. Requirement of correction was based on the expertise of the interventional radiologist, where particular focus was placed on obtaining a locally optimized registration such that anatomical landmarks (e.g. cysts, renal cortex, local vasculature) close to the area of interest were best matched. Hereafter, SAFIR automatically derived the tumor size defined as the maximum 3D diameter of the tumor and the minimal treatment margin (MTM) defined as the smallest 3D distance between the tumor and ice-ball surface rounded to the nearest millimeter, where negative values indicates incomplete coverage. The side of the tumor where the smallest margin or sides where suspected incomplete coverage occurred were documented by dividing the tumor in octants along the left-right, cranio-caudal, and anterior-posterior axes.

### Statistical analysis

The primary endpoints were MTM and LTP during follow-up. For statistical analysis, SPSS (version 25.0) was used. Continuous data are expressed as means ± standard deviation or medians with interquartile range (IQR) or range. Baseline parameters were compared between subgroups using independent T-test for normally distributed variables and Mann-Whitney U test for non-normally distributed variables. Fisher’s exact test and Fisher-Freeman-Halton-test were used for categoric variables with respectively two or more than two possible outcomes. Correlation between tumor size and MTM was analyzed using Spearman’s rho test. The effect of tumor size and MTM on local outcome were evaluated using univariable Cox proportional hazards regression model and presented as hazard ratios with corresponding 95% confidence intervals. A *P* value < 0.05 was considered statistically significant.

## Results

### Demographics

A total of 32 cryoablation procedures were performed for 32 renal tumors in 20 males (66.6%) and 10 females (33.4%). Median age was 69 years (range, 38–83 y). Mean tumor diameter was 3.1 ± 0.9 cm. Tumor size was significantly larger in cases with LTP compared to local control (4.1 ± 0.5 vs. 2.9 ± 0.9 cm, *P* = .003) (Table 2). No significant differences between the groups were found in age, sex, ASA classification and RENAL score.

**Table 2:** Distribution of baseline parameters between cases with local control and local tumor progression

	Local control (***N*** = 26) No (%), mean ± SD or median (range)	Local tumor progression (***N*** = 6) No (%), mean ± SD or median (range)	***P***-value
Age (years)	71 (38–83)	72 (58–79)	0.507
Sex			0.647
Male	17 (65.4)	3 (50.0)	
Female	9 (34.6)	3 (50.0)	
Maximal tumor diameter (mm)	29.1 ± 0.9	41.0 ± 0.5	**0.003**
ASA classification			0.194
II	17 (65.4)	2 (33.3)	
III	9 (34.6)	4 (66.6)	
RENAL score			0.070
Low	18 (69.2)	2 (33.3)	
Moderate	8 (31.8)	3 (50.0)	
High	0 (0)	1 (16.7)	

### Clinical outcome

Local control was achieved in 26 tumors (81%). LTP occurred in 6 tumors (19%), of which two showed residual disease and four showed recurrent disease. 1- and 2-year LTP-free survival rates were 90.1 and 76.8%, respectively. Pretreatment biopsy report of the cases with LTP showed RCC in 4 patients (67%), oncocytoma or mixed oncocytoma/chromophobe RCC in 1 patient (17%) and was inconclusive in 1 patient (17%). The median follow-up was 16 months (range, 1–58 months). The median time to tumor recurrence was 17 months (range, 6–23 months). Both patients with residual disease and three of four patients with recurrent disease underwent repeated treatment with MR-guided cryoablation. The fourth patient with local recurrence died due to metastatic disease, which was already present at the time of cryoablation.

Major complications (Clavien-Dindo ≥3) occurred in two (6.1%) cases. Urosepsis (grade 3b) requiring a double-J catheter under general anesthesia occurred in one patient, and sepsis and postrenal obstruction requiring short term dialysis at the Intensive Care Unit (grade 4a) occurred in one patient.

### Procedural results

Overall, a median of 3 cryoprobes per patient were used (range, 2–4). The mean ice ball volume was 51.9 ml (range, 16.3–115.4 ml). Ice ball volume and number of cryoprobes did not differ significantly in cases with vs. without LTP. The mean tumor volume was 9.8 ml (range, 1.0–39.4 ml). Tumor volume was significantly higher in cases with versus without LTP (15.5 ± 5.3 ml vs. 8.5 ± 8.8, *P* = .003).

### Minimal treatment margin

**Fig. 3:**
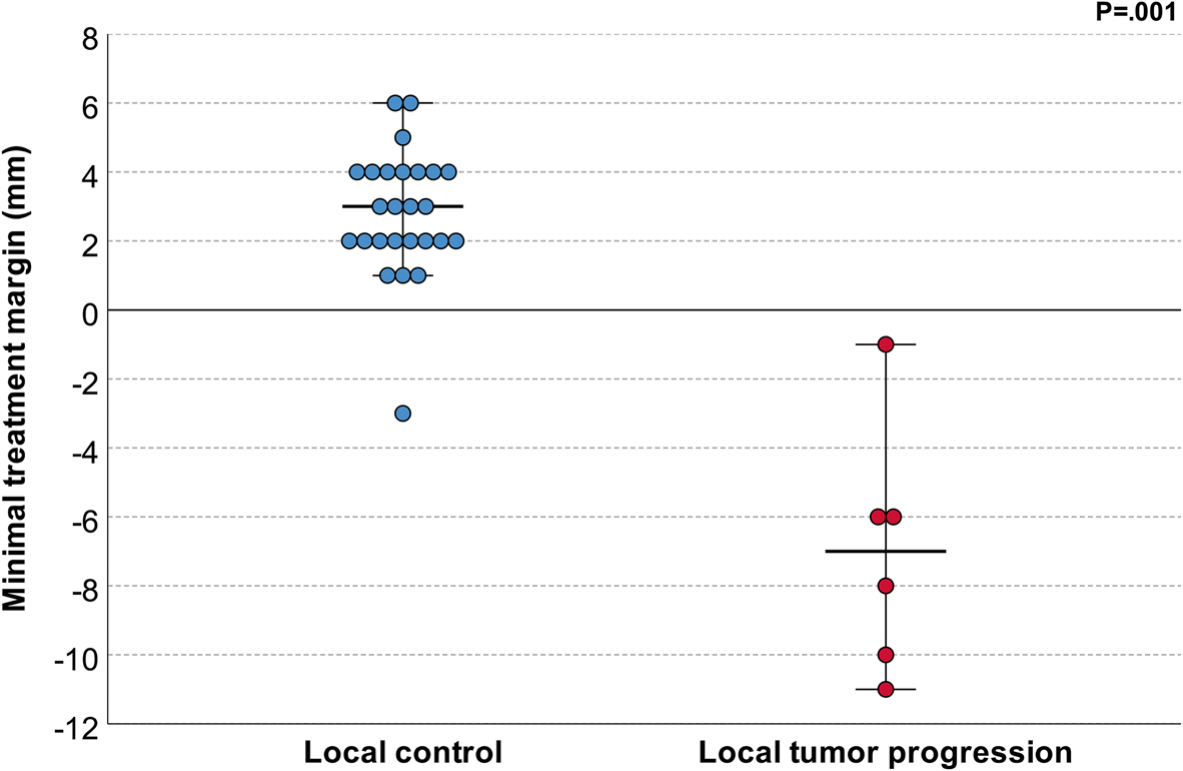
Minimal treatment margin for cases with local tumor control and local tumor progression

**Fig. 4:**
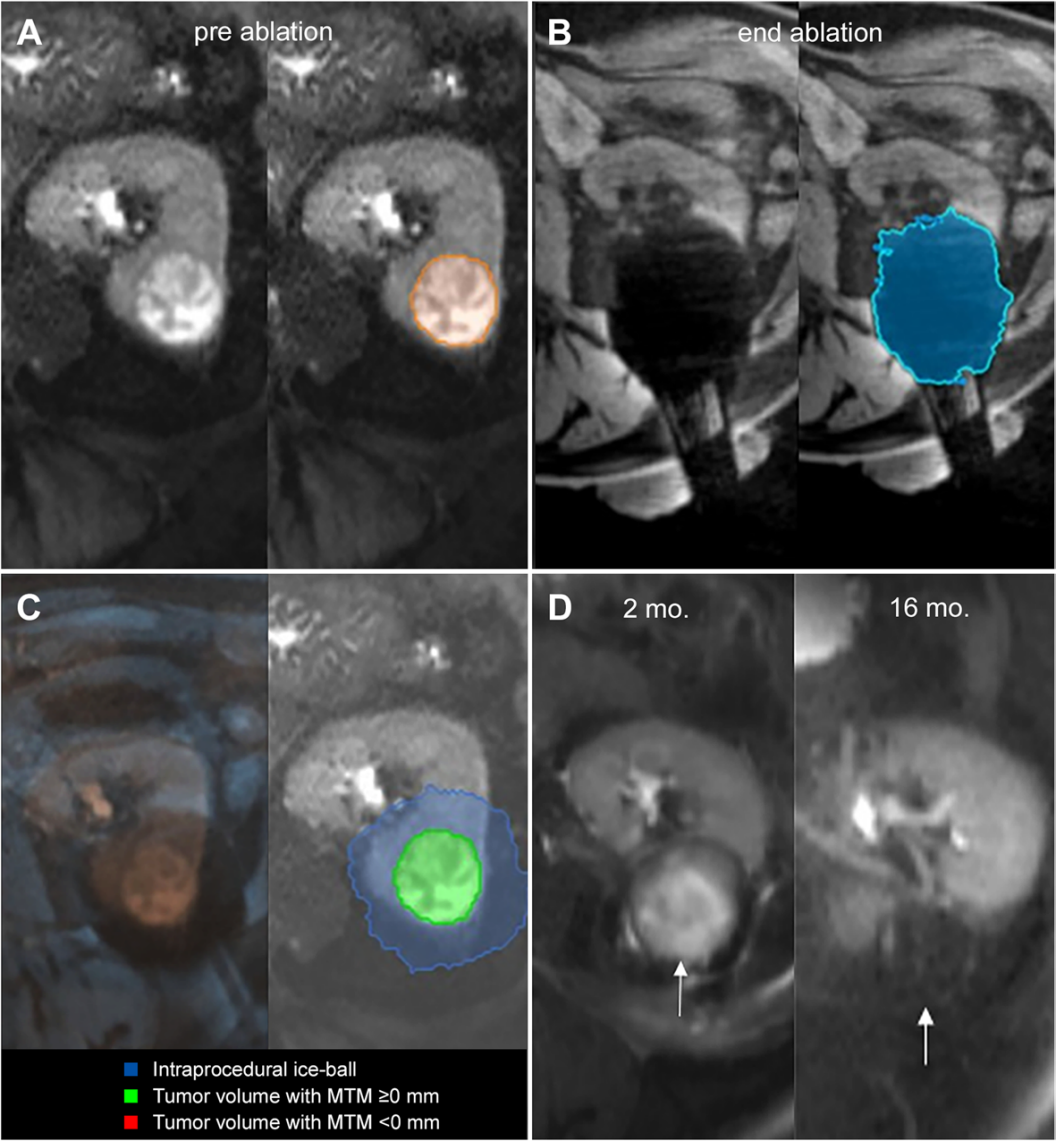
**A** Segmentation (orange outline) of a 3.5 cm diameter tumor located interpolar in the left kidney on pre-ablation axial T2-weighted MR imaging. **B** Segmentation (blue outline) of corresponding end ablation ice-ball volume on post-ablation axial T1-weighted MR imaging. **C** Overlay of pre-ablation and post-ablation images after co-registration (left) and resulting 3D margin calculation (right). Volumetric assessment shows complete tumor coverage (green) by the end-ablation ice-ablation ice-ball (blue) with MTM of 4 mm. **D** Ablation zone (white arrows) without tumor recurrence on follow-up axial T2-weighted MR imaging 2 months (left) and 16 months (right) after cryoablation

**Fig. 5:**
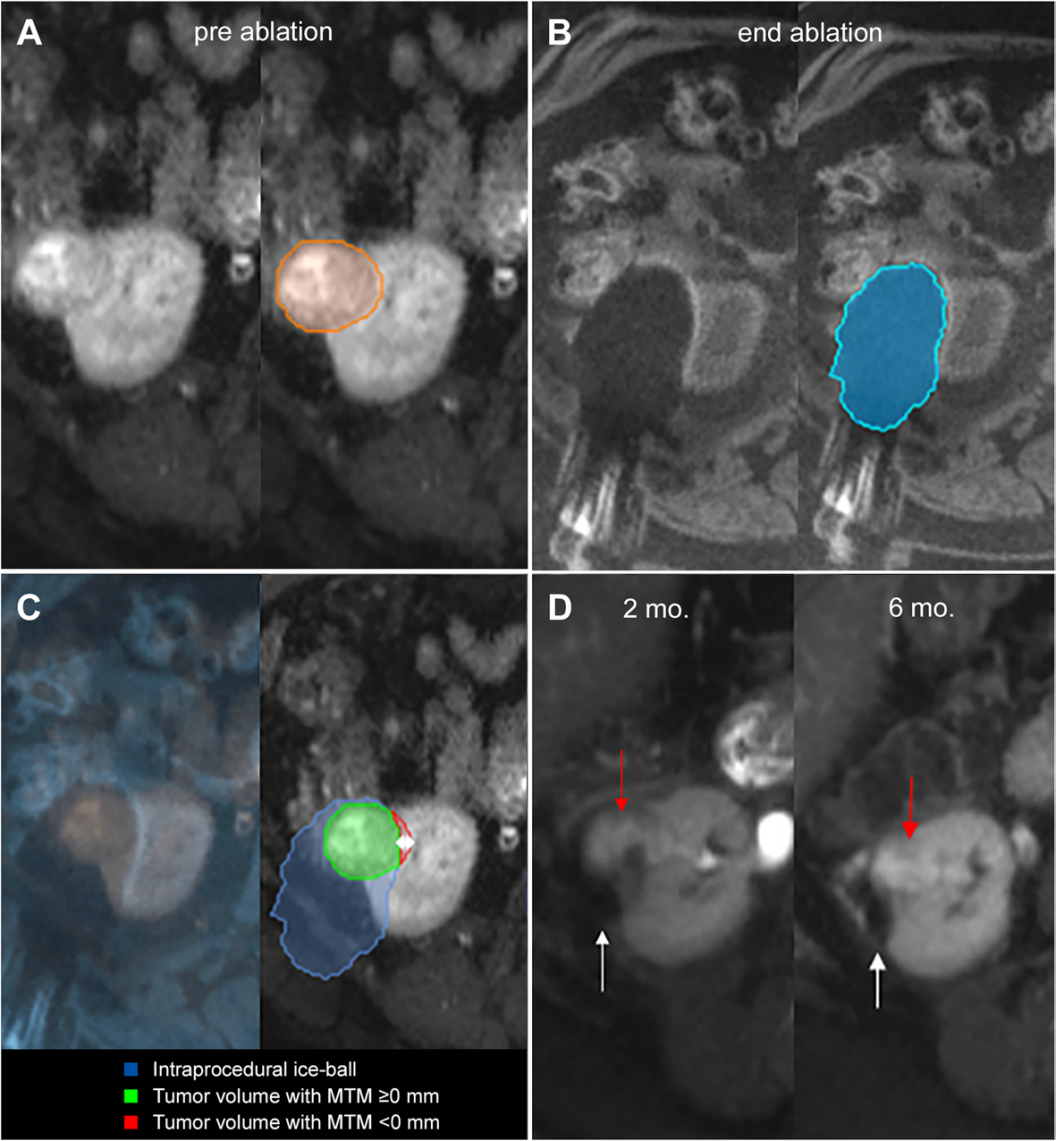
**A** Segmentation of a 4.2 cm diameter tumor located in the lower pole of the right kidney (outlined in orange) on pre-ablation axial T2-weighted MR imaging. **B** Segmentation of the corresponding ice ball (outlined in blue) on post-ablation axial T1-weighted MR imaging. **C** Overlay of pre-ablation and post-ablation images after co-registration (left) and resulting 3D margin calculation (right). Volumetric assessment shows predicted incomplete ablation at medial side (red) with MTM of − 6 mm. **D** Follow-up axial T2-weighted MR imaging at 2 months (left) after cryoablation shows ablation zone (white arrows) with suspected incomplete coverage on medial side (red arrows). Six months follow-up MRI (right) confirms local tumor progression (red arrow) at the medial side

The mean MTM was 1.0 ± 4.4 mm (range, − 11 to 6 mm). The intended treatment margin extending ≥5 mm beyond the tumor border in all directions was achieved in 3/32 cases (9,4%). Median MTM was significantly smaller for cases with LTP compared to cases without LTP (− 7 mm, IQR:-10 to − 5 vs. 3 mm, IQR: 2 to 4 mm; *P* = .001) (Fig. 3). Representative cases of local control and local tumor progression after cryoablation are shown in Fig. 4 and 5, respectively.

All (6/6) cases of LTP had a negative MTM. Moreover, in all six (100%) patients with LTP, the site of tumor regrowth matched with the side of the smallest treatment margin. One patient showed a negative MTM and did not show LTP on follow-up imaging. All cases with an MTM ≥1 mm showed local control.

**Fig. 6:**
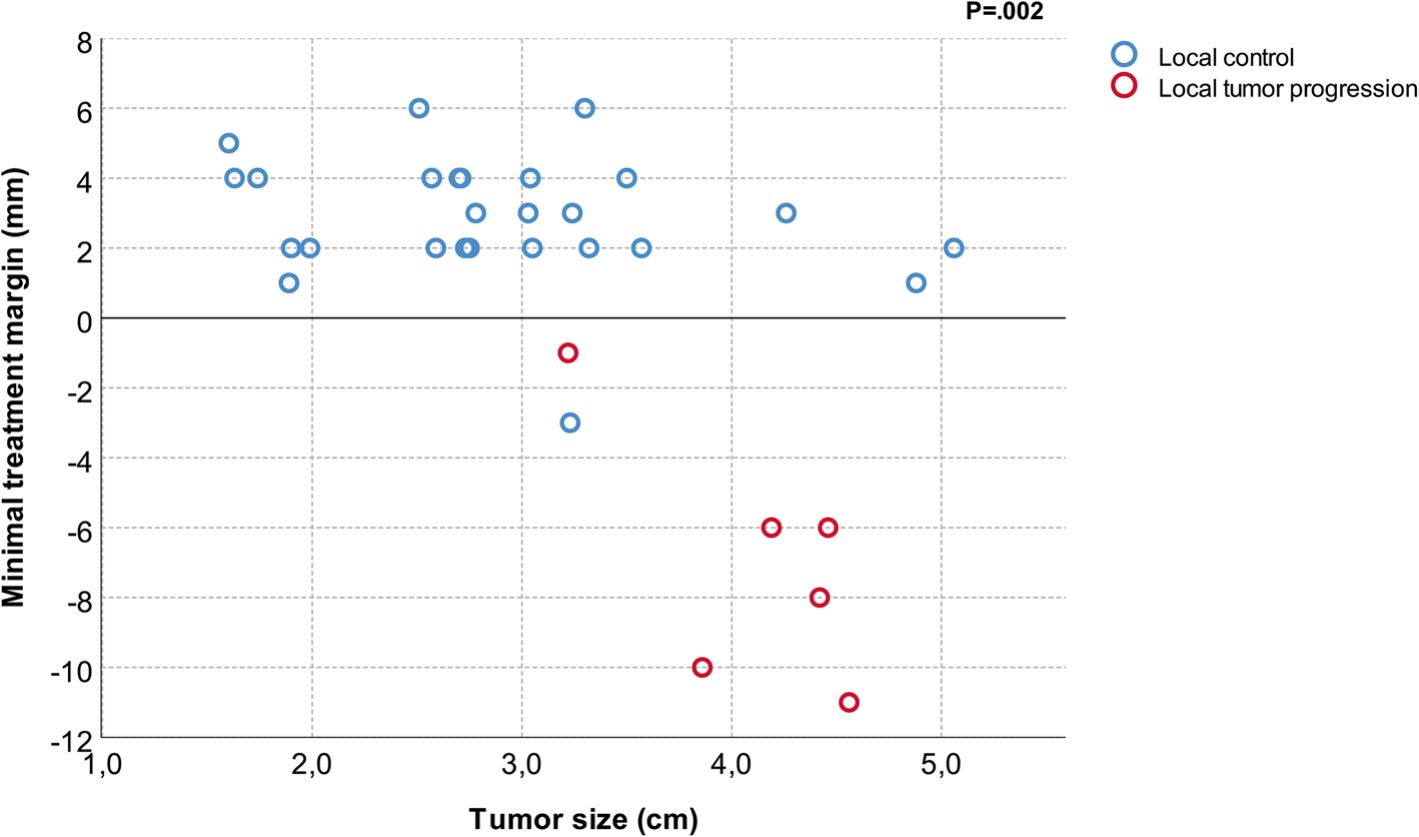
Minimal treatment margin plotted against tumor size for cases of local control and local tumor progression

On univariable Cox’s analysis, minimal treatment margin (*P* < .001) and tumor size (*P* = .012) were significantly associated with local tumor control (Table 3). An increase in tumor size was associated with smaller treatment margins (*P* = .002) (Fig. 6). All treatment margins < 0 mm occurred in patients with tumor size ≥3 cm.

**Table 3:** Cox regression analysis of possible predictors for local tumor progression

	Hazard ratio (95% Cl)	***P***-value
Minimal treatment margin	0.77 (0.66–0.89)	< 0.001
Maximal tumor diameter	3.56 (1.32–9.57)	0.012

## Discussion

In this study, we demonstrate feasibility of deriving volumetric treatment margins from intraoperative pre- and post-ablation MRI which appear correlated to local outcome after renal cryoablation. In our preliminary clinical data all cases of local tumor progression were associated with negative treatment margins, corresponding to predicted incomplete ablation coverage. Cases treated with an intraoperative MRI-derived minimal margin extending at least 1 mm beyond the MRI-visible tumor led to local control and this was more difficult to achieve in tumors > 3 cm.

In clinical practice a treatment margin of 5 mm is aimed for to ensure radical treatment during cryoablation of small renal tumors [10, 12–14]. However, two recent studies described a treatment margin below 5 mm to be adequate for local tumor control. Ge et al. [15] included 63 patients treated with PCA under CT-guidance and reported treatment margins on 3D reformatted axial intraprocedural CT images. They concluded a treatment margin ≥1.5 mm had highest overall accuracy in predicting successful ablation. Fraisse et al. [16] reported on 117 patients who underwent PCA under CT-guidance. They determined treatment margins on MR images made 1 day postoperative and concluded that treatment margins < 1 mm are associated with higher recurrence rate. These data are in line with our results, as we observed that a volumetric margin ≥5 mm was only achieved in 9% of cases without leading to high incidence of local recurrence. In part, these findings may be due to the ability to assess the full treatment margin in 3D. Where an overall safety margin of 5 mm can be considered safe, we are now able to detect that in some areas a smaller margin may have occurred without leading to local tumor recurrence. In addition, cryoablation is known to cause direct thermal damage as well as delayed cytotoxic effects, mainly due to thrombosis of blood vessels causing local ischemia [19]. These effects may not yet be captured on the intraprocedural MRI scan visualizing the total ice extent, and possibly contribute to a smaller intraprocedurally determined treatment margin to be sufficient for complete ablation.

A notable finding was that one patient with a negative treatment margin did not show local tumor progression during follow-up. Intraprocedural biopsy showed clear cell RCC histology. One-month follow-up MRI scan showed unsharp delineation of the ablation zone without residual enhancement, diffusion restriction or specific tumor characteristics, uncertain for residual disease. However, further follow-up imaging with contrast enhanced CT-scans did not confirm this finding. This patient was already diagnosed with metastasized RCC which was stable at the time of the cryoablation. However, systematic therapy with a tyrosine kinase inhibitor (pazopanib) was initiated 1.5 years after the cryoablation. This may have masked any potential tumor progression to date. In addition, one patient with an oncocytoma showed evident local tumor recurrence on follow-up imaging. This is unexpected for a tumor type considered to have a benign behavior and may suggest presence of a malignant component. On secondary review of the biopsy, a hybrid oncocytoma-chromophobe lesion [20] could neither be definitively verified nor excluded on histopathology.

Our study supports previous literature suggesting an association between tumor size and increased risk of local tumor progression. Our univariate analysis shows each 1-cm increase in tumor size provides a 3.56-fold increased risk of local tumor progression. Although conflicting results are described in previous literature [21], recent studies showed that tumor size is a predictor for local tumor recurrence [22–24]. In a recent study by Pickersgill et al. [25] including 296 T1a and 32 T1b tumors (mean tumor size 2.7 cm), tumor size was a significant predictor of disease progression with a hazard ratio of 1.32 per 1-cm increase in size.

Our results also indicate tumor size to inversely correlate with treatment margin. We hypothesize this contributes to the higher tumor progression rates in larger tumors, since tumors with smaller treatment margins have a higher risk for inadequate treatment and local tumor progression. Possible explanations for the smaller treatment margins in larger tumors are more caution during the treatment to avoid damage of surrounding structures or the overestimation of the synergistic effect of multiple needles used causing a smaller ice ball than expected. Our observations suggest that treatment margins should be evaluated with extra caution in tumors > 3 cm to avoid undertreatment.

Although not a primary outcome of this study, we observed a 90.1% 1-year and 76.8% 2-year local recurrence-free survival, which is somewhat lower than previously reported in literature. Zondervan et al. [13] reviewed six studies, each including > 50 patients who underwent PCA with a follow-up between 20 and 36 months. The 2-year RFS was > 95% in four studies and the 3-year RFS was 86.0 and 83.6% in the remaining studies. However, their review included only T1a (< 4 cm) renal tumors while our study also included seven T1b (≥4 cm) renal tumors. Since only few studies have been performed on cryoablation for T1b renal tumors, a wide range of 1-year RFS rates has been reported (60–97%) [26–28]. In the current study 4 out of 6 patients with tumor progression had a T1b tumor, suggesting these tumors have a relatively large contribution to the overall RFS rates.

Our study has several limitations, including a single-institutional retrospective design, limited sample size and intermediate term follow-up. Fundamental issues related to the MR technique may influence the assessment of the tumor margin, including the spatial resolution of MRI and possible motion artefacts. Image registration was used to correct any in-between scan displacement and although care was taken to locally optimize the registration to the treatment area, minor errors in image registration can influence accuracy of our results. Another limitation is the difficulty of obtaining consistency in manual segmentation of the tumor and ice ball as well as MR image fusion, where only a single interventional radiologist reader was included in this study. Therefore larger, prospective studies are warranted to validate our findings and come to a minimal safe threshold that may be useful to confirm treatment margins intraoperatively. Sporadically, patients with contraindications to undergo MR-guided cryoablation were treated under CT guidance and were not included in the study cohort. Furthermore, the absence or inconclusiveness of biopsy in some patients presents a diagnostic difficulty in clinical practice and may have introduced a bias to the cohort. Also, the lack of routinely performed biopsy to histologically confirm presence of residual or recurrent disease is a matter of debate. Clinically, local tumor progression is diagnosed based on established radiographic criteria [29].

## Conclusions

In conclusion, this study shows that volumetric assessment of treatment margins derived from intraoperative MR images is feasible and can be useful to predict renal cryoablation margin status. The intended treatment margin > 5 mm was often not achieved in practice. In our preliminary data an intraoperative MRI-derived minimal margin extending at least 1 mm beyond the MRI-visible tumor led to local control and this was more difficult to achieve in tumors > 3 cm. Finally, online 3D analysis of achieved treatment margins may be a valuable tool to intraoperatively assess therapy success, but larger, prospective studies are needed to establish a safe threshold for clinical use.

## Data Availability

The datasets used and/or analysed during the current study are available from the corresponding author on reasonable request.

## Abbreviations

3D: Three-dimensional
CT: Computed tomography
IQR: Interquartile range
LTP: Local tumor progression
MRI: Magnetic resonance imaging
MTM: Minimal treatment margin
PCA: Percutaneous cryoablation
RCC: Renal cell carcinoma
RFS: Recurrence-free survival
SAFIR: Software assistant for interventional radiology
